# Anomeric modification of carbohydrates using the Mitsunobu reaction

**DOI:** 10.3762/bjoc.14.138

**Published:** 2018-06-29

**Authors:** Julia Hain, Patrick Rollin, Werner Klaffke, Thisbe K Lindhorst

**Affiliations:** 1 Christiana Albertina University of Kiel, Otto Diels Institute of Organic Chemistry, Otto-Hahn-Platz 3–4, D-24118 Kiel, Germany, Fax: +49 431 8807410; 2 Université d’Orléans et CNRS, ICOA, UMR 7311, BP 6759, 45067 Orléans, France, Fax: +33 238 417281; 3 Haus der Technik e.V., Hollestr. 1, 45127 Essen, Germany, Fax: +49 201 1803269

**Keywords:** anomeric stereoselectivity, carbohydrates, glycoside synthesis, Mitsunobu reaction

## Abstract

The Mitsunobu reaction basically consists in the conversion of an alcohol into an ester under inversion of configuration, employing a carboxylic acid and a pair of two auxiliary reagents, mostly triphenylphosphine and a dialkyl azodicarboxylate. This reaction has been frequently used in carbohydrate chemistry for the modification of sugar hydroxy groups. Modification at the anomeric position, leading mainly to anomeric esters or glycosides, is of particular importance in the glycosciences. Therefore, this review focuses on the use of the Mitsunobu reaction for modifications of sugar hemiacetals. Strikingly, unprotected sugars can often be converted regioselectively at the anomeric center, whereas in other cases, the other hydroxy groups in reducing sugars have to be protected to achieve good results in the Mitsunobu procedure. We have reviewed on the one hand the literature on anomeric esterification, including glycosyl phosphates, and on the other hand glycoside synthesis, including S- and N-glycosides. The mechanistic details of the Mitsunobu reaction are discussed as well as this is important to explain and predict the stereoselectivity of anomeric modifications under Mitsunobu conditions. Though the Mitsunobu reaction is often not the first choice for the anomeric modification of carbohydrates, this review shows the high value of the reaction in many different circumstances.

## Introduction

Fifty years ago, Oyo Mitsunobu reported a preparation of esters from alcohols and carboxylic acids supported by two auxiliary reagents, diethyl azodicarboxylate (DEAD) and triphenylphosphine [1]. This reaction has ever since become known as the “Mitsunobu reaction”, being a frequently utilized tool in organic synthesis. In 1981, Mitsunobu published a first review about this reaction, entitled "The Use of Diethyl Azodicarboxylate and Triphenylphosphine in Synthesis and Transformation of Natural Products" [2]. Thereafter, several further general reviews have appeared [3–6], owing to the spectacular development of diversified synthetic applications of the Mitsunobu reaction, whilst the long term debate about the mechanism of this reaction was still ongoing [7–12].

**Scheme 1:**
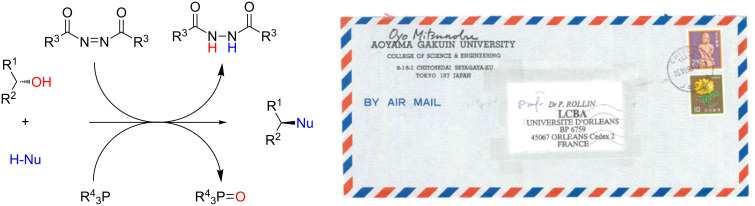
Left: The Mitsunobu reaction is essentially a nucleophilic substitution of alcohols occurring with inversion of configuration at the alcohol stereocenter. The auxiliary reagents are involved in a redox process. Right: Original correspondence with Professor Oyo Mitsunobu (letter to P.R. in 1993).

The standard Mitsunobu reaction involves coupling of an alcohol and a nucleophile in a dehydrative S_N_2 process activated by a reactive combination of a triaryl- or trialkylphosphine as reducing agent and a dialkyl azodicarboxylate as oxidant. In a redox process, the phosphine species is oxidized to the respective phosphine oxide and the azo reagent is reduced to the corresponding 1,2-hydrazinodicarboxylate (Scheme 1). As we have frequently utilized this valuable reaction in carbohydrate chemistry, in this account we have compiled literature, where the Mitsunobu reaction was used for the anomeric modification of carbohydrates.

The reaction proceeds under mild, neutral conditions that are compatible with a wide range of functional groups. In the case where a stereogenic center is involved, the reaction takes place with stereochemical inversion [6]. The reaction partners are mostly primary or secondary alcohols, while the nucleophilic species needs to be acidic [13] with a p*K*_a_ < 11. Otherwise the azo reagent would compete with the acidic nucleophile and participate in the substitution reaction [14]. Various compounds comply with that condition: carboxylic acids, phenols, hydrazoic acid, some other NH acids, and thiols. The standard azo reagents used are diethyl- (DEAD) or diisopropyl- (DIAD) azodicarboxylate. However, alternative reagents such as azodicarboxamides [15–16] or stabilized phosphoranes were also developed to allow reaction with nucleophiles of weaker acidity. The typical phosphine reagents are triphenyl- (Ph_3_P) or tributylphosphine (*n*-Bu_3_P). In recent years, advances have been made using solid supported reagents, thus facilitating work-up conditions [17–18]. The polarity of the commonly aprotic solvents used in the Mitsunobu reaction, including toluene, tetrahydrofuran or dimethylformamide, has been shown to be influential in terms of efficacy and stereoselectivity [19].

Since its infancy, the Mitsunobu reaction has found applications in carbohydrate chemistry, as its broad scope and mild conditions are ideal for the formation of conjugates with sensitive natural products. Standard applications of the Mitsunobu reaction in glycochemistry have mostly dealt with the functionalization of the primary hydroxy group of sugars and, to a lesser extent, with modifications of the secondary alcohol array in carbohydrate rings [2–6], for example for halogenation [20]. However, the Mitsunobu reaction can also be profitably utilized for the anomeric modification of carbohydrates. Hence, we have focused this review on the utilization of the Mitsunobu reaction for manipulations of the carbohydrate hemiacetal, where reducing (anomerically unprotected) sugars react as the alcohol component to be either converted into glycosides or into other anomerically modified carbohydrate derivatives. We intend to provide a critical survey as well as a source of inspiration, even more so as glycosylation remains a challenge in carbohydrate chemistry.

## Review

### Mechanistic considerations

**Scheme 2:**
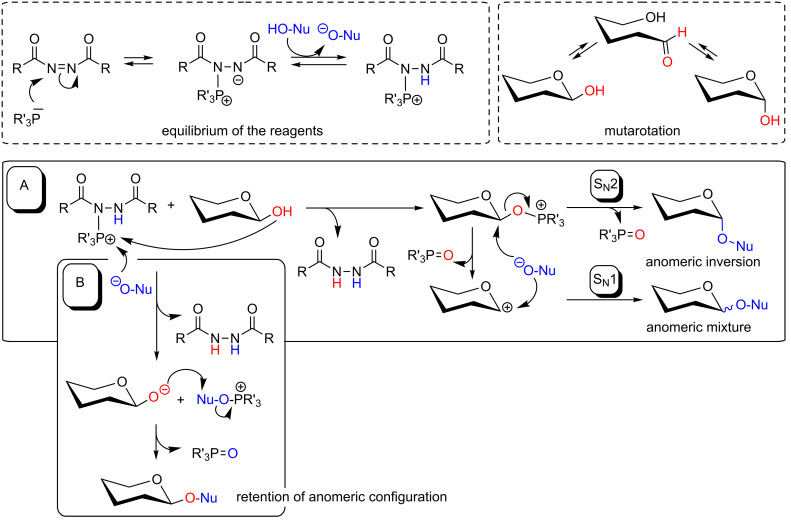
Mechanistic considerations on the Mitsunobu reaction with carbohydrate hemiacetals (depicted in simplified form). Two equilibria are relevant for this reaction (top dashed boxes), (i) the formation of the “Mitsunobu reagent” from the employed azodicarboxylate, phosphine and the acidic nucleophile, and (ii) the mutarotation equilibrium of the reducing sugar in solution. These can give rise to at least two different reaction pathways, A and B, as explained in the main text. Depending on various parameters, the anomerically modified sugar, a glycoside or an anomeric ester, respectively, is obtained with full inversion of anomeric configuration or as anomeric mixture (A), or with retention of the anomeric configuration via *O*-alkylation (B). For clarity both reaction pathways are exemplified with only one sugar anomer.

Since Mitsunobu’s postulate of a three-reaction-step mechanism in 1981 [2] many further mechanistic investigations have been performed and reported [3,7–8,19]. To rationalize the outcome of the Mitsunobu reaction with reducing sugars, special mechanistic considerations have to be taken into account. On the one hand, the equilibrium between the azodicarboxylate, the phosphine, and the acidic component, Nu-OH, is important (cf. Scheme 2, left dashed box). On the other hand, mutarotation of the sugar hemiacetal has to be discussed to predict the stereochemical outcome of the reaction. Mutarotation results in an equilibrium of both, α- and β-anomers (Scheme 2, right dashed box). However, full anomerization is often not observed as the rate and the extent of mutarotation depends on various parameters such as anchimeric effects of neighboring groups and the reaction conditions. Hence it has been frequently observed in Mitsunobu reactions with carbohydrate hemiacetals, that sugar anomerization is either absent or slower than the formation of the *O*-glycosyloxyphosphonium salt, which can play the intermediate during the reaction (Scheme 2, pathway A) [21]. Another possible explanation for limited anomerization lies in the different stability of anomeric glycosyloxyphosphonium salts, where one anomer can be sterically favored over the other, thereby pushing the equilibrium to a product with the respective anomeric configuration. Regardless of the rate of mutarotation, the Mitsunobu reaction can proceed through a mechanistic pathway A or B as depicted in Scheme 2. Especially when the sugar alcohol is not sterically hindered, phosphorus transfer occurs to yield a phosphine-activated anomeric alcohol (a glycosyloxyphosphonium ion, pathway A). This in turn can be attacked by the deprotonated nucleophile resulting in an anomerically modified carbohydrate with inversion of configuration at the anomeric center, according to a S_N_2 mechanism. Pathway A can also proceed through a S_N_1 mechanism when the intermediate glycosyloxyphosphonium ion is less stable. Then, it can decompose into the corresponding anomeric oxocarbenium ion and phosphine oxide. The oxocarbenium ion would then react with the NuO^−^ anion in a S_N_1 mechanism. While this would lead to racemization under normal circumstances, in most carbohydrates, participation effects of neighboring groups in the vicinity (typically at the 2-position of the sugar ring) affect the reaction outcome, favoring nucleophilic attack from a preferred face of the sugar ring [22–23]. Grynkiewicz and colleagues have discussed anchimeric assistance even when no protecting group is present at C-2, assuming a Brigl’s anhydride type intermediate [24]. In the absence of a substituent at C-2, however, typically poor stereoselectivity is observed in Mitsunobu reactions with carbohydrate hemiacetals, indicating a S_N_1-type pathway A of the reaction [25].

The Mitsunobu reaction can also follow a different pathway B (Scheme 2), as first suggested by Hughes [13] and later by Ahn et al. [26]. Assuming that the alcohol is sterically hindered and thus represents a relatively weak nucleophile, the deprotonated acidic partner, NuO^−^, can react with the phosphonium intermediate first to afford an intermediate Nu-O-PR’_3_. In the case where a carboxylic acid is used, Nu-O-PR’_3_ represents an acyloxyphosphonium ion. This in turn reacts with the anomeric oxyanion to furnish the anomerically modified sugar with retention of configuration via anomeric *O*-alkylation. This mechanistic proposal is in agreement with observations by Lubineau et al., who could correlate the acidity of the employed nucleophile with the anomeric outcome of the Mitsunobu reaction [27].

Both reaction pathways, A and B, have a “raison d’être” in addressing different outcomes of the Mitsunobu reaction, which vary depending on the substrates used. While these variables make the already complex Mitsunobu reaction even more demanding, they can also be manipulated to one’s advantage, for example for the stereoselective formation of β-mannosides [28].

### Reactions with protic acids to achieve anomeric esters

**Scheme 3:**
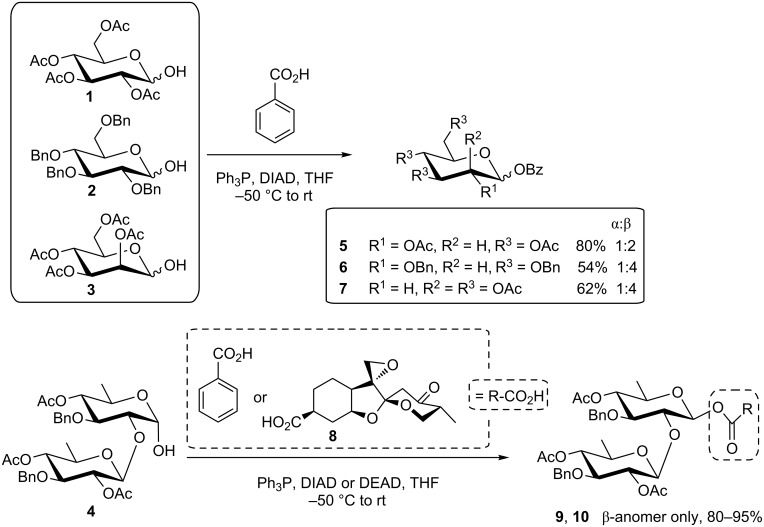
Anomeric esterification using the Mitsunobu procedure [29].

The first application of the Mitsunobu reaction involved esterification of a secondary alcohol. Although an anomeric OH group cannot be regarded as a classical secondary alcohol group but as a hemiacetal OH, it can be successfully involved in Mitsunobu reactions to achieve 1-*O*-acyl glycoses. Thus, searching for an efficient protocol for the preparation of complex, multifunctional glycosyl esters in the context of the total synthesis of phyllanthostatin antitumor agents, A. B. Smith and colleagues soundly investigated the suitability of the Mitsunobu reaction [29]. They concluded already back in 1986 that “the anomeric hydroxyl group of various pyranose hemiacetals can be esterified with inversion of configuration, conveniently, mildly and on large-scale using Ph_3_P, with either DIAD or DEAD and a carboxylic acid in THF at either –50 °C or at room temperature”. Hence, several protected mono- and disaccharides, such as **1**–**4** (Scheme 3) were selectively esterified with simple benzoic acid to give **5**–**7** and **9**, respectively. In addition, **4** was also converted with the phyllanthostatin aglycone **8** to give **10** with inversion of anomeric configuration. Extension of this work to other more complex antineoplastic glycosyl esters was successfully investigated by the same group [30–34].

**Scheme 4:**
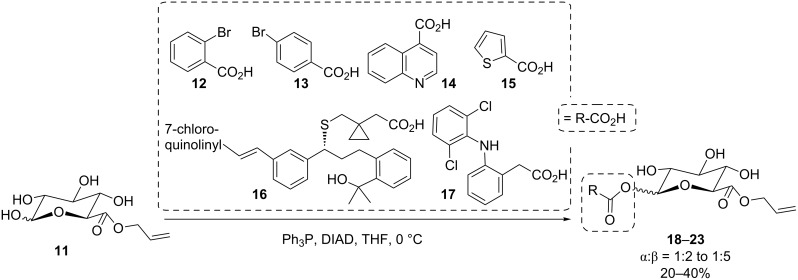
Conversion of allyl glucuronate into various 1-O-esterified allyl glucuronates using anomeric Mitsunobu esterification [36–37].

De Mesmaeker et al. reported the stereoselective coupling of an allyl glucuronide, in which all hydroxy groups except the anomeric OH were *O*-acyl-protected, with carboxylic acids by a Mitsunobu reaction [35]. The reaction was successful even when a free phenolic function was present in the employed acid and the desired β-anomer of the 1-*O*-acyl-β-D-glucuronide products could be isolated in up to 50% yield. Similarly, regioselective esterification of unprotected allyl glucuronide **11** was performed by Juteau et al. with the acids **12**–**16** yielding anomeric mixtures of the respective 1-*O*-acyl-β-D-glucuronides **18**–**22** in quite acceptable yields even with complex acids like **16** (Scheme 4) [36]. The same approach was chosen in the Stachulski group for the anomeric modification of glucuronides with the anti-inflammatory drug diclofenac (**17**) to give the respective product **23** (Scheme 4) [37].

Bourhim et al. reported that the Mitsunobu reaction with native D-glucose, D-GlcNAc or D-maltose resulted in regioselective esterification of the primary OH group, leaving all other hydroxy groups including the anomeric OH unmodified [38]. On the other hand, other authors have reported that the anomeric position can be selectively modified in a Mitsunobu reaction without concomitant modification of the primary 6-OH (vide infra). Apparently, fine-tuning of reaction conditions can alter the selectivity of the Mitsunobu reaction and in addition, different regioselectivities might origin in the structure of the sugar substrate.

**Scheme 5:**
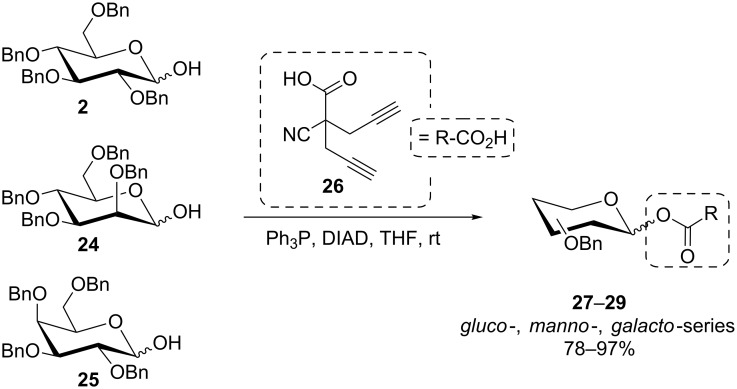
Synthesis of anomeric glycosyl esters as substrates for Au-catalyzed glycosylation [40].

In the course of a synthesis of carbocyclic lignan variants related to podophyllotoxin, a pseudo-anomeric stereospecific inversion of a carbasugar was achieved in good yield in Nishimura’s group [39]. More recently, the Mitsunobu procedure was applied in the context of gold-catalyzed glycosylation in order to install a reactive anomeric ester function in a series of *O*-benzylated glycoses (**2**, **24**, **25**) employing the branched carboxylic acid **26** (Scheme 5) [40]. The produced esters **27**–**29** were obtained as anomeric mixtures.

**Scheme 6:**
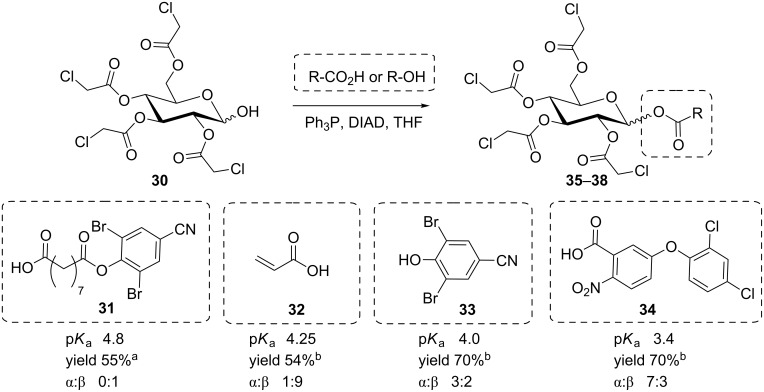
Correlation between p*K*_a_ value of the employed acids (or alcohol) and the favoured anomeric configuration of the respective product. ^a^Carried out at 0 °C; ^b^carried out at rt [27].

Lubineau et al. [27] investigated the stereoselectivity of the anomeric Mitsunobu coupling of 2,3,4,6-tetra-*O*-chloroacetyl-D-glucose (**30**) as well as its *galacto*-configured analogue with the carboxylic acids **31**–**34** to obtain products **35**–**38** which are related to various pesticide agents (Scheme 6). Their results supported the theory that, along with an effect of the reaction temperature, an increase of the p*K*_a_ of the employed acidic reaction partner can lead to predominant formation of the β-configured product, whereas stronger acidic reagents can favor the formation of the respective α-anomers. These findings can be explained by considering the two different reaction pathways A and B as shown above in Scheme 2. The authors state that the observed p*K*_a_ effect is either due to the influence of the acidity of the employed acid on the reaction mechanism or results from the proton-catalyzed change of the anomeric ratio of the starting material **30** in solution.

**Scheme 7:**
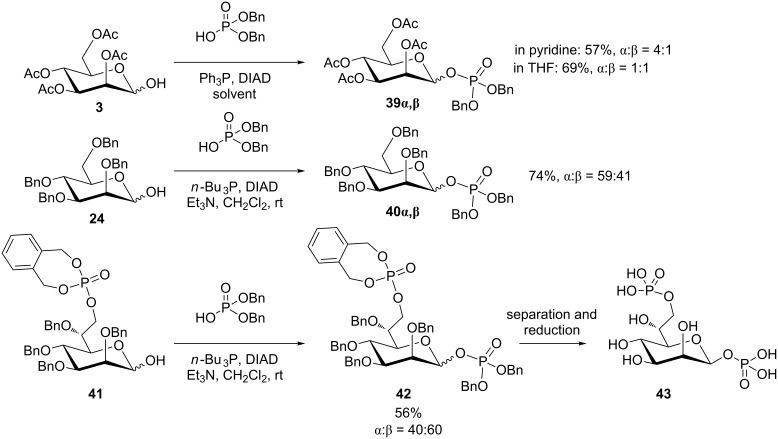
Synthesis of the β-mannosyl phosphates for the synthesis of HBP **43** by anomeric phosphorylation according to Mitsunobu [41–42].

Very recently, anomeric phosphorylation via a Mitsunobu approach was concomitantly undertaken by groups from Japan and Austria, respectively [41–42], aiming at the synthesis of the bacterial metabolite and potent innate immune modulator D-*glycero*-β-D-*manno*-heptose-1,7-bisphosphate (**43**, HBP, Scheme 7). The group around Zamyatina employed 2,3,4,6-tetra-*O*-acetyl-mannopyranose (**3**) as a 9:1 α,β-mixture in order to optimize the reaction conditions for the Mitsunobu reaction with phosphoric acid dibenzyl ester. The anomeric mannosyl phosphate derivatives **39α** and **39β** were obtained in 57% total yield when pyridine was used as the solvent, as depicted in Scheme 7. In THF, the same reaction furnished a 1:1-anomeric mixture in 69% yield. The authors thus considered the Mitsunobu reaction as unsatisfactory for the synthesis of HBP. On the other hand, Inuki et al. optimized the Mitsunobu conditions with 2,3,4,6-tetra-*O*-benzyl-mannopyranose (**24**) and found that the addition of trimethylamine in dichloromethane improved the Mitsunobu process, leading to **40α** and **40β** in more than 70% yield. When such optimized conditions were applied to the mannose-6-phosphate derivative **41**, the desired bisphosphate **42** was obtained in 56% yield as a 40:60 α,β-anomeric mixture before work-up, and in a 53:47 ratio after work-up due to slight anomerization. As **42** can be easily converted into the target molecule, the authors concluded, that in spite of the poor stereoselectivity, the Mitsunobu reaction constitutes a key step in a successful access to β-mannosyl phosphates such as **43**.

### Reactions with phenols to achieve aryl glycosides

**Scheme 8:**
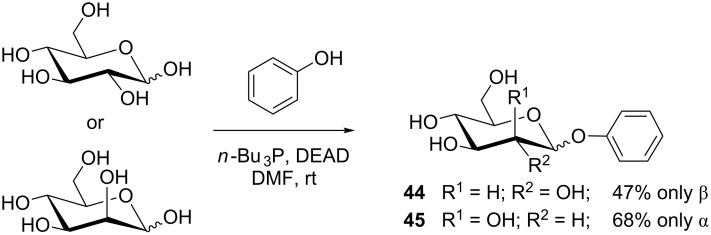
Synthesis of phenyl glycosides **44** and **45** from unprotected sugars [24].

Not only anomeric esters, but also glycosides can be obtained through the Mitsunobu reaction. Dehydrative glycosylation approaches with reducing sugars were previously reviewed [43–44]. As phenols are weak acids, they are suitable reaction partners in the Mitsunobu reaction, leading to aryl glycosides with reducing sugars as the alcohol components. Grynkiewicz can be called the pioneer of Mitsunobu glycosylation, as having explored the Mitsunobu reaction for the synthesis of various aryl glycosides [24,45]. Thus, native sugars such as D-glucose and D-mannose (Scheme 8) were converted into the respective unprotected phenyl glycosides **44** and **45** with phenol in just one step in moderate to good yields.

**Scheme 9:**
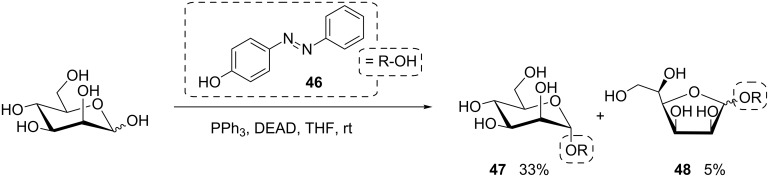
Synthesis of azobenzene mannosides **47** and **48** without protecting group chemistry [46].

Recently, the scope of this synthetic approach was expanded by the Lindhorst group employing D-mannose and hydroxyazobenzene **46** for the synthesis of the photoswitchable azobenzene α-D-mannoside **47** (Scheme 9) [46]. Notably, in this reaction, traces of an anomeric mixture of the respective furanoside **48** were detected.

**Scheme 10:**
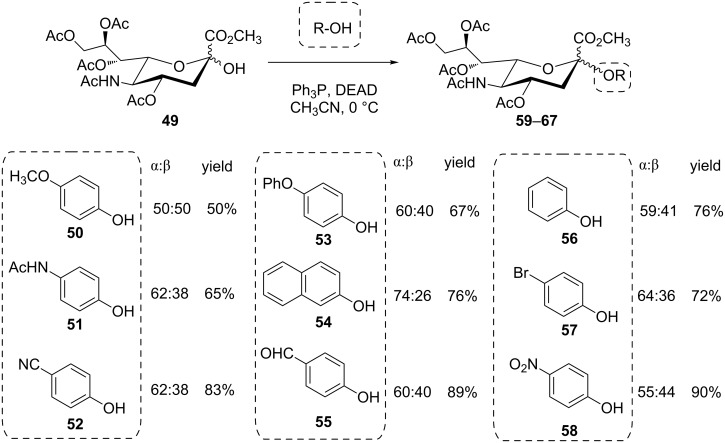
Synthesis of various aryl sialosides using Mitsunobu glycosylation [25].

The Mitsunobu synthesis of aryl glycosides was also applied to *p*-nitrophenol [47], naphthols [48–49], or multifunctional phenols [27,50]. Such arylglycosylation was also extended for the synthesis of aureolic acid antibiotics [21,51–52]. In search of convenient methods for the synthesis of aryl sialosides, Gao et al. explored the scope of the Mitsunobu reaction with the sialic acid derivative **49**, employing a range of phenols **50**–**58** in acetonitrile to achieve sialosides **59**–**67**, albeit with modest anomeric selectivity (Scheme 10) [25].

Interestingly, no correlation between the p*K*_a_ of the employed acids and the stereoselectivity of the reaction could be established in this case, since similar anomeric mixtures were obtained throughout all experiments. In this case, the absence of a neighboring group in position 3 of the sugar ring could account for low stereoselectivity. To explain the lack of stereoselectivity, the authors considered a S_N_1 reaction mechanism, involving the respective oxocarbenium ion or, alternatively, the formation of both α- and β-configured glycosyloxyphosphonium ions, which are in turn displaced by the nucleophile in the expected S_N_2 fashion, resulting in a respective anomeric mixture of products (cf. Scheme 2).

In contrast to this, the yields of the obtained aryl sialosides strongly correlated with the p*K*_a_ of the utilized phenols, with stronger acids leading to higher yields. This yield-to-p*K*_a_ correlation is in accordance with earlier findings in the synthesis of aryl glucuronides where the yields were equally affected by the p*K*_a_ of the chosen phenols, while the neighboring group effect was found to govern the stereochemical outcome of the reaction towards β-configured products [53]. In this case, phenolic chromium tricarbonyl complexes of weaker acids such as *p*-cresol were employed to improve the yield.

**Scheme 11:**
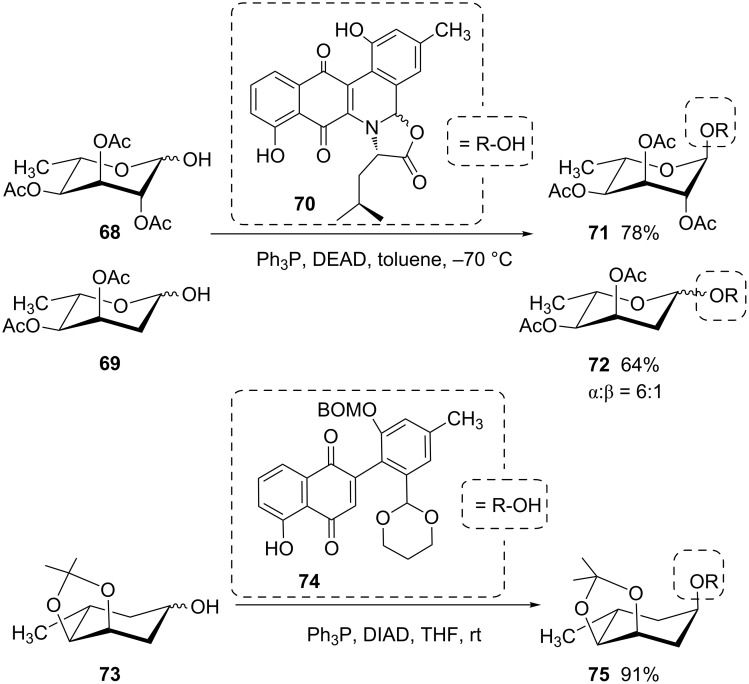
Mitsunobu synthesis of different jadomycins [54–55]. BOM: benzyloxymethyl.

The challenge of glycoside synthesis using sugars devoid of a C-2 participating group is also highlighted by a total synthesis of various jadomycins [54]. Whereas the Mitsunobu glycosidation of **68** with the phenolic aglycon **70** yields the pure 1,2-*trans*-glycoside **71**, the 2-deoxy sugar **69** yields the glycoside **72** as a 6:1 α,β-anomeric mixture (Scheme 11). In contrast to this, the jadomycin B carbasugar analogue **75** was formed stereoselectively from the 2-deoxy-carbasugar **73** in a Mitsunobu reaction with the aglycon **74** [55].

**Scheme 12:**
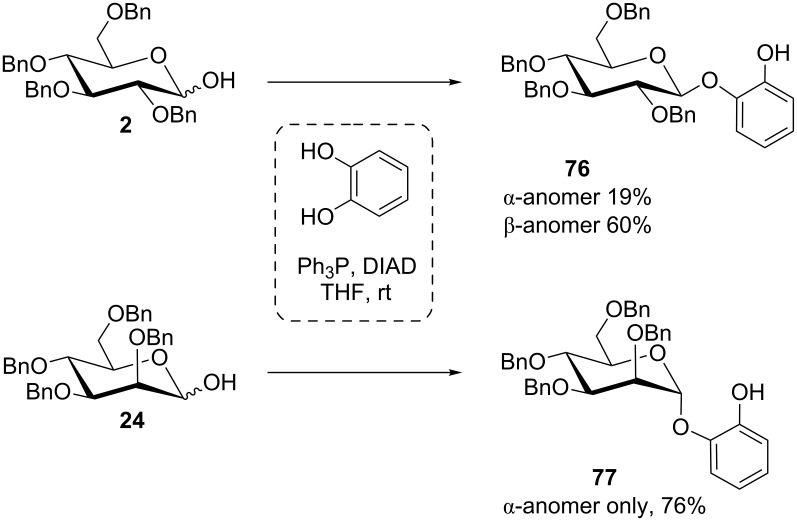
Stereoselectivity in the Mitsunobu synthesis of catechol glycosides in the *gluco*- and *manno*-series [56].

Benzyl protection, which does not exert neighboring group effects in classical glycosylations, resulted in the predominant formation of 1,2-*trans* glycosides in the Mitsunobu reaction with catechol. In fact, benzyl-protected reducing glucose derivative **2** gave the β-glucoside **76** with good stereoselectivity, and the respective mannose derivative **24** resulted in the pure α-mannoside **77** in good yield (Scheme 12) [56].

**Scheme 13:**
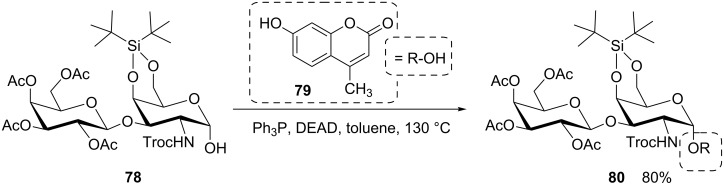
Formation of a 1,2-*cis* glycoside **80** assisted by steric hindrance of the β-face of the disaccharide through the DTBS protection. DTBS: di-*tert*-butylsilylene; Troc: 2,2,2-trichloroethoxycarbonyl [22].

In a general approach to coumarin-derived inhibitors of gyrase B, a group working at Hoechst Marion Roussel developed a Mitsunobu process to connect noviose with a broad range of 7-hydroxycoumarins [57]. Similarly, Imamura and colleagues used 4-methylumbelliferone (**79**) as acidic reaction partner in a Mitsunobu glycosylation with a reducing galabioside **78** (Scheme 13) [22]. Advantage was taken of the bulky DTBS protecting group to enforce α-stereoselection despite of the anchimeric effect of the vicinal *N*-Troc protecting group to achieve the α-glycoside **80** in high yield. Nevertheless, this reaction needed optimization, such as an unusually high reaction temperature.

**Scheme 14:**
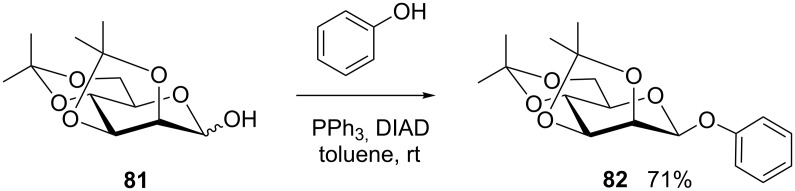
Stereoselective β-D-mannoside synthesis [60].

Also weakly acidic phenols were used by Vaccaro et al. [58] for Mitsunobu glycosylation in the D-glucuronic series, employing the reagent pair *n*-Bu_3_P-ADDP (1,1’-(azodicarbonyl)dipiperidine) developed by Tsunoda et al. [59]. Interestingly, Davis and co-workers could employ 2,3:4,6-di-*O*-isopropylidene mannopyranose **81** in a Mitsunobu reaction with phenol to stereoselectively achieve the respective β-mannoside **82** in good yield (Scheme 14) [60].

This stereo-differentiating effect of isopropylidene protecting groups was also observed in other cases with D-mannopyranose [46,61]. It might be used as a key to a reliable approach to otherwise difficult to synthesize β-mannosides using the Mitsunobu procedure. This approach to 1,2-*cis*-mannosides is equally effective when cyclohexylidene protecting groups are used [28,47,62].

**Scheme 15:**
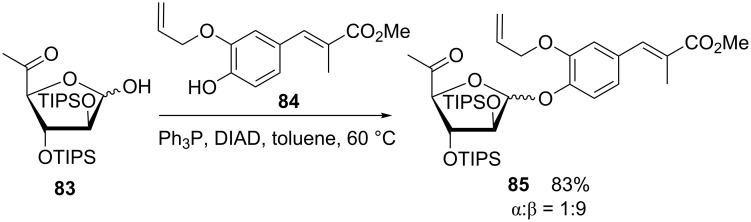
TIPS-assisted synthesis of 1,2-*cis* arabinofuranosides [63]. TIPS: triisopropylsilyl.

Mitsunobu glycosylation was also a successful method in total synthesis. In the course of a 17-step synthesis of hygromycin A, Donohoe et al. used a Mitsunobu glycosylation of **84** with the arabinose derivative **83**. This reaction could be tuned to deliver the required β-arabinofuranoside building block **85** with high stereoselectivity and under the assistance of triisopropylsilyl (TIPS) protecting groups (Scheme 15) [63].

Similar reaction conditions were applied by Nie et al. in the total synthesis of the nucleoside antibiotic A201A [64]. Notably, the *n*-Bu_3_P-ADDP reagent system led here to the formation of the pure α-glycoside. Likewise, a Mitsunobu glycosylation of complex phenols was successfully implemented in the preparation of novobiocin analogues [65], and formed a key step in the synthesis of new glycosidic PDE4 (phosphodiesterase type 4) inhibitors [66]. Also calix[4]arenes could be selectively mono- or diglycosylated by means of the Mitsunobu methodology [67–68].

**Scheme 16:**
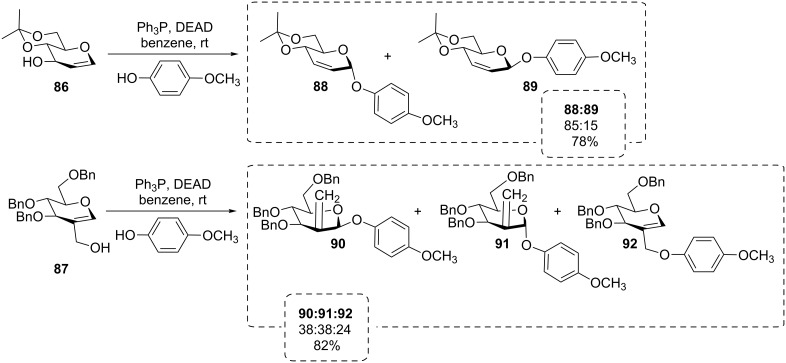
The Mitsunobu reaction with glycals leads to interesting rearrangement products [69].

The Mitsunobu reaction was also employed with glycals like **86** and **87** reacting with *p*-methoxyphenol as an alternative to the Ferrier rearrangement in the synthesis of 2-*C*-methylene glycosides and other rearrangement products **88**–**92**, some of which cannot be obtained in a classical Ferrier reaction (Scheme 16) [69–72]. The results outlined in Scheme 16 are consistent with early findings of Guthrie et al. exploring the Mitsunobu benzoylation of 4,6-*O*-benzylidene-D-allal [73].

### Reactions with alcohols to yield alkyl glycosides

**Scheme 17:**
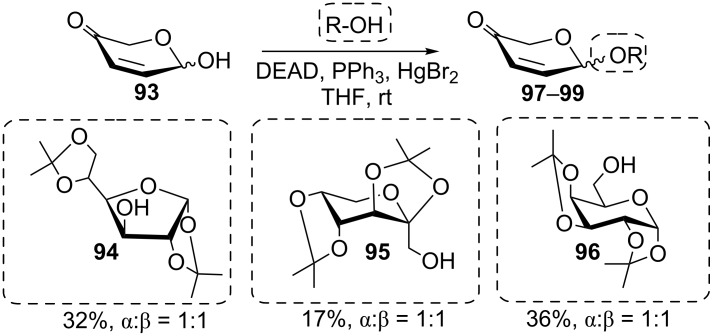
Synthesis of disaccharides using mercury(II) bromide as co-activator in the Mitsunobu reaction [75].

In contrast to aryl ethers, the formation of alkyl ethers is not observed under Mitsunobu conditions. Likewise, standard alcohols are typically poor reaction partners in Mitsunobu glycosylations. Due to their high p*K*_a_ values, the formation of the transient phosphonium betaine is hampered [43]. In an effort to overcome this drawback, several decades ago, Szarek et al. tested mercuric halides to assist the betaine formation in such cases, and indeed cyclohexyl glycosides could be formed in various sugar series with decent yields [74]. Consequently, this approach was explored in a Mitsunobu-type disaccharide synthesis reacting **93** with the alcohol components **94**–**96** to give **97**–**99**, albeit with moderate success (Scheme 17) [75].

**Scheme 18:**
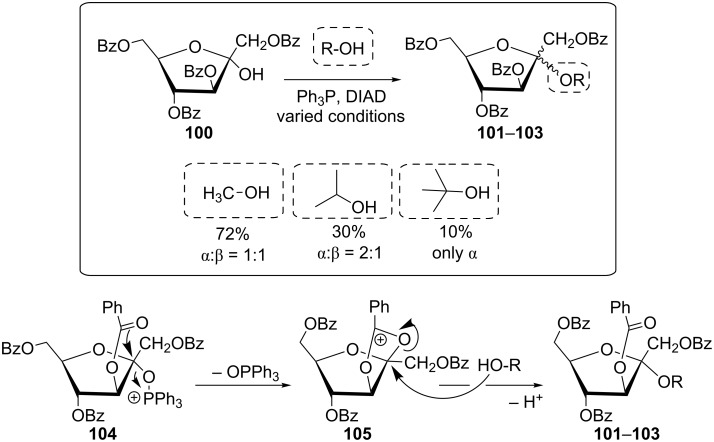
Synthesis of various fructofuranosides according to Mitsunobu and proposed neighbouring group participation [23].

Contradictory results were reported on the Mitsunobu glycosylation of 1,3,4,6-tetra-*O*-protected fructofuranosides. In contrast to Guthrie et al. [76], Bouali and colleagues claimed an effective synthesis of alkyl fructofuranoside **101**–**103** from **100** using simple alcohols (Scheme 18) [23]. The reaction was rationalized by participation of the C-3 neighboring group (structure **104**) with intermediate formation of a dioxolanium derivative **105** [23].

**Scheme 19:**
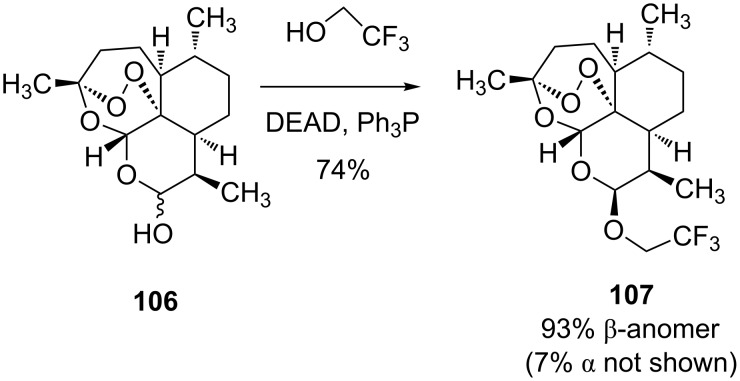
The Mitsunobu reaction allows stereoslective acetalization of dihydroartemisinin [77].

On the other hand, the Mitsunobu reaction was advantageous for the acetalization of the antimalarial drug dihydroartemisinin **106** to give **107** with trifluoroethanol, having a p*K*_a_ of 12.4 (Scheme 19) [77]. The efficiency of the Mitsunobu glycosylation with fluorinated alcohols with p*K*_a_ values between 9 and 12 was demonstrated with several other examples [78].

**Scheme 20:**
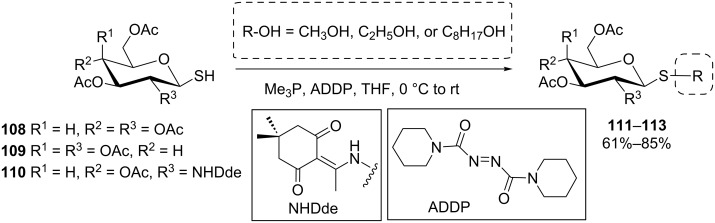
Synthesis of alkyl thioglycosides by Mitsunobu reaction [81].

Also thiols, according to their p*K*_a_ value range between 10 and 11 should be qualified appropriate reagents for a Mitsunobu thioglycosylation. However, a competitive redox reaction with the PR_3_-azodicarboxylate reagent system precludes this application [79–80]. In spite of that, thioglycosides **111**–**113** could be prepared via a Mitsunobu-type condensation of thioglycosides such as **108** and **109** with simple alcohols (Scheme 20) [81–82]. In this case, of course, the sugar thioglycoside takes the role of the nucleophile rather than of the alcohol component in the Mitsunobu reaction.

### Reactions with NH acids to achieve N-glycosides

**Scheme 21:**
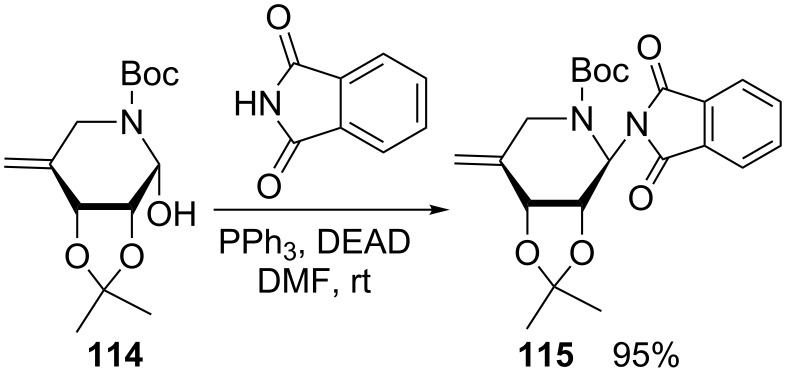
Preparation of iminoglycosylphthalimide **115** from **114** [85].

Early on, phthalimide was regarded as a good Mitsunobu reagent, owing to its NH acidity with a p*K*_a_ of 8.3, thus offering the opportunity for the synthesis of N-glycosides of the *N*-glycosylimide type [83]. However, along with the formation of *N*-glycosylphthalimides, a side-reaction takes place, producing both glycosyl carbonates and *N*-glycosyl-1,2-dialkoxycarbonylhydrazines [84]. This anomeric *N*-phthalimidation was later implemented by Nishimura et al. for the iminosugar **114** with phthalimide to give **115** in a high yield, en route to a new family of α-L-fucosidase inhibitors (Scheme 21) [85].

**Scheme 22:**
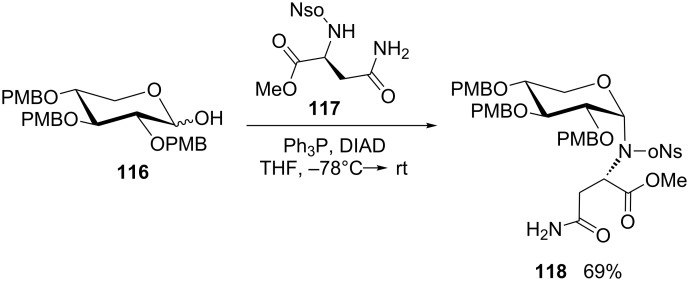
Mitsunobu reaction as a key step in the total synthesis of aurantoside G [87].

More generally, the preparation of modified glycosylamines under Mitsunobu conditions requires a sufficiently acidic NH nucleophile. A particularly illustrative procedure was disclosed by van Boom’s group, who used *N*-nosyl-activated amino-acid esters for anomeric modification of sugars in order to produce substrates for a novel route to Amadori rearrangement products [86]. The same approach was recently adopted in a total synthesis of aurantoside G, involving the Mitsunobu ligation of a D-xylopyranose derivative **116** and *N*-nosylated methyl asparaginate **117** to give **118** (Scheme 22) [87].

**Scheme 23:**
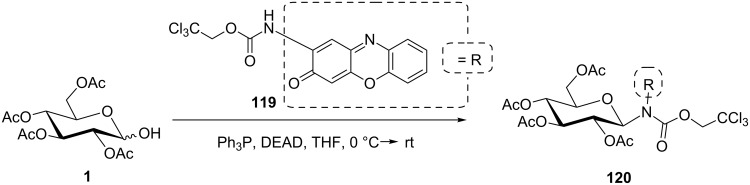
Utilization of an N–H acid in the Mitsunobu reaction [88].

Compared to *N*-sulfonylation, *N*-carbamoylation can also prove effective to enhance the acidity of a NH group. Hence, the trichloroethoxycarbonyl (Troc) protection/activation of the amino group of questiomycin **119** allowed Igarashi et al. to access the *N*-glucosylated derivative **120** in good yield and complete β-stereoselectivity from hemiacetal **1** (Scheme 23) [88].

**Scheme 24:**
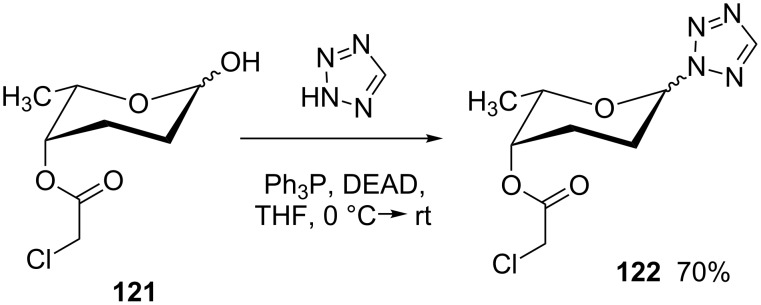
Mitsunobu reaction with 1*H*-tetrazole [89].

Also some aza-heterocycles bearing a free NH group possess a low enough p*K*_a_ to allow Mitsunobu coupling. In the course of the synthesis of the hexasaccharidic fragment of landomycin A, the L-rhodinose derivative **121** underwent glycosylation with 1*H*-tetrazole to give **122**, which has a p*K*_a_ that compares to carboxylic acids (Scheme 24) [89].

**Scheme 25:**
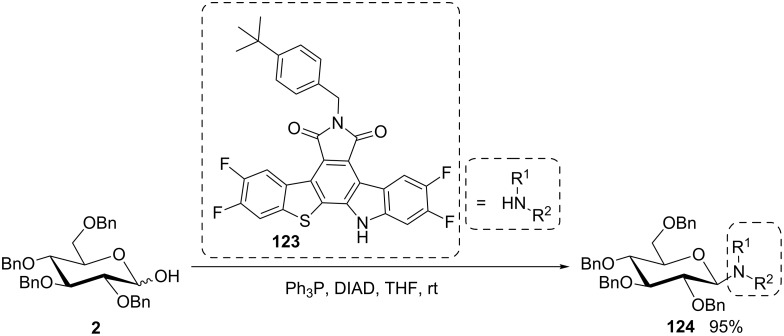
Formation of a rebeccamycin analogue using the Mitsunobu reaction [101].

In spite of the fact that parent indole is too weak an acid to undergo Mitsunobu conversions, a model maleimide–indole hybrid was investigated by Ohkubo and colleagues to pave the way for the synthesis of indolo[2,3-*a*]pyrrolo[3,4-*c*]carbazole compounds with anticancer activity [90–91]. N-Glycosides of indole derivatives were also approached by Zembower et al. employing 2,3,4,6-tetra-*O*-benzyl glucopyranose in a Mitsunobu reaction [92]. In the same period, Prudhomme’s group followed closely related approaches for the *N*-glycosylation of indolic structures. Various rebeccamycin analogues were efficiently synthesized from indolo[2,3-*c*]carbazole frameworks using the methodology previously developed by Voldoire et al. [93]. Further applications to 7-aza-indolic analogues of rebeccamycin [94–96], granulatimide and isogranulatimide [97–100] were also reported. In addition, using the same Mitsunobu methodology, the rebeccamycin analogue **124** was synthesized in high yield and complete β-stereoselectivity by Wang et al. from the glucose derivative **2** and **123** (Scheme 25) [101].

Application of the anomeric Mitsunobu coupling in nucleoside synthesis was pioneered by Szarek et al. [102], who reacted 6-chloropurine with various reducing sugars using methyldiphenylphosphine as activator. Extension to the D-*ribo* series with 6-chloro- and 2,6-dichloropurines was later reported by Hertel and co-workers [103]. In the course of an exploration of modified L-nucleosides, 6-chloropurin-9-yl derivatives were obtained in moderate yields [104]. Aiming at an improved procedure to synthesize nucleosides with glycosylation of the nucleobase, De Napoli et al. used the Bu_3_P-ADDP system to connect inosine and uridine derivatives with D-ribofurano and D-glucopyrano moieties [105]. Hocek’s group in 2015 published a direct one-pot synthesis of exclusively β-configured nucleosides from unprotected or 5-*O*-monoprotected D-ribose using optimized Mitsunobu conditions with various purine- and pyrimidine-based heterocycles. Here, DBU was applied first, followed by DIAD and P(*n*-Bu)_3_ [106]. Two years later Seio and colleagues set out to systematically study the effect of phosphine, azodicarbonyl reagent, and solvent on the yield and α/β ratio in the synthesis of 2'-deoxynucleosides [107]. They reported that the highest yield and β-selectivity were obtained using (*n*-Bu)_3_P and 1,1′-(azodicarbonyl)dipiperidine in DMF. In a model study directed towards the synthesis of guanofosfocin, Sugimura et al. used the Mitsunobu *N*-glycosylation to attach a glucopyranosyl donor on either 6-*N*-trityl-8-oxoadenosine or 6-*O*-benzyl-8-oxoinosine [108].

### Reactions with N–OH acids to yield NO-glycosides

**Scheme 26:**
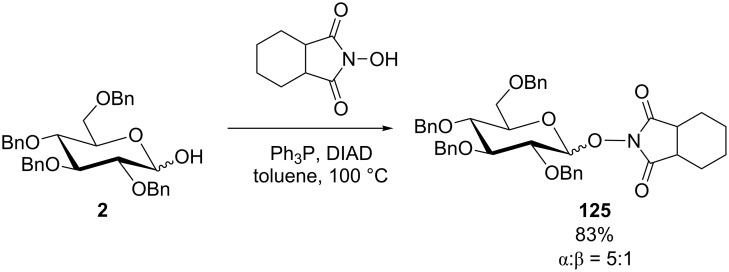
Synthesis of carbohydrates with an alkoxyamine bond [114].

Because of its well-suited p*K*_a_ (6.3), *N*-hydroxyphthalimide was early considered in Mitsunobu reactions, for example by Grochowski and Jurczak to form an anomeric phthalimide–oxy bond as shown in several sugar series [109–111]. This gives access to new *O*-glycosylhydroxylamines, namely for the construction of glycosidic N–O linkages in calicheamycin oligosaccharides [112–113]. This option was applied in the synthesis of trichostatin D involving glucose derivative **2** and *N*-hydroxyhexahydrophthalimide as the glycosyl acceptor to give **125** (Scheme 26) [114].

By using diverse *N*-hydroxylated azaheterocycles in the Mitsunobu glycosylation, Grochowski explored the synthesis of new nucleoside analogues. 1-Hydroxy-benzotriazole, 1-hydroxy-2-cyanobenzimidazole, 1-hydroxyuracil, and 1-hydroxythymine were used to prepare the respective NO-furanosides in the *manno*- and *ribo*-series [115–117].

### Miscellaneous

**Scheme 27:**
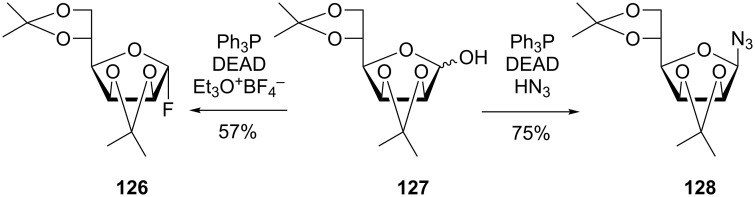
Synthesis of glycosyl fluorides and glycosyl azides according to Mitsunobu [118–119].

The Mitsunobu reaction was also applied for other anomeric modifications, such as fluorination, reported by Kunz et al. for the synthesis of the α-D-mannofuranosyl fluoride **126**, however, in moderate yield (Scheme 27) [118]. The advantage of this approach lies in the mild fluorine source, triethyloxonium tetrafluoroborate, which, in combination with the Ph_3_P-DEAD system, leaves the acid-labile protecting groups of **127** intact, other than when HF is used. Zbiral’s group on the other hand, developed the synthesis of glycosyl azides such as **128** in a Mitsunobu procedure with **127**, using hydrazoic acid as the azide source (Scheme 27) [119].

This approach was extended by Besset et al. to D-fructose and a range of unprotected mono- and disaccharides, again showing a preference of the reaction for the anomeric position instead of the primary [120]. Anomeric azidation was also investigated on diverse unprotected hexopyranoses by Larabi et al. using a modified Appel-type procedure [121].

**Scheme 28:**
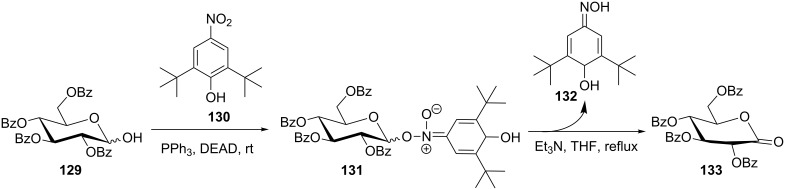
Anomeric oxidation under Mitsunobu conditions [122].

A striking oxidation reaction of alcohols to carbonyl compounds was disclosed by Mitsunobu and colleagues, involving the sterically hindered nitrophenol **130** [122]. With sugars like **129**, the Mitsunobu glycosylation is hampered, and instead an anomeric aci-nitroester **131** is formed, which is converted into the corresponding gluconolactone **133** under elimination of a quinone monoxime **132** (Scheme 28).

## Conclusion

In this account, 15 years after Professor Mitsunobu has passed away, we have surveyed the literature on the Mitsunobu reaction for anomeric modifications of carbohydrates. As in classical glycosylation reactions, not all mechanistic details of the anomeric conversion of sugars in a Mitsunobu process are known and well understood. Hence until today, surprising results and unexpected side reactions are being observed in Mitsunobu type conversions of hemiacetals. In addition, the reaction conditions of a Mitsunobu process often require particular optimization efforts. Thus, the Mitsunobu reaction has not become a standard procedure in glycoside synthesis nor in anomeric esterification, but on the other hand, it was demonstrated to serve as a key step in many cases of carbohydrate modification including total synthesis of sensitive natural products. This is also due to the mild and neutral conditions under which the Mitsunobu reaction occurs. Additionally, it has a rather broad scope as many building blocks are acidic enough to react with reducing sugars representing the alcohol component of the reaction. The stereochemical outcome of a Mitsunobu glycosylation is often advantageous such as in the synthesis of β-D-mannosides, which are otherwise difficult to prepare. However, often, the stereoselectivity of the reaction is less definite than our text books claim. Unfortunately, the Mitsunobu reaction is uneasy to scale up and this is probably one of the biggest obstacles for a broad and also technical use of this reaction. Nevertheless, this review proves that in the glycosciences, the Mitsunobu reaction must not be overlooked as it is an important method in the synthetic toolbox for anomeric modification of sugars and glycoconjugate preparation.
